# Pediatric Acute Q Fever Mimics Other Common Childhood Illnesses

**DOI:** 10.1371/journal.pone.0088677

**Published:** 2014-02-10

**Authors:** Ingeborg Y. Bart, Yvonne Schabos, Roeland W. N. M. van Hout, Alexander C. A. P. Leenders, Esther de Vries

**Affiliations:** 1 Department of Pediatrics, Jeroen Bosch Hospital’s-Hertogenbosch, The Netherlands; 2 Department of Linguistics, Radboud University, Nijmegen, The Netherlands; 3 Department of Medical Microbiology, Jeroen Bosch Hospital's-Hertogenbosch, The Netherlands; University of North Carolina School of Medicine, United States of America

## Abstract

Knowledge of Q fever has increased over the last decades, but research has mainly focused on adults. Data in children are scarce, and current knowledge is mostly based on case reports. The aim of this study was to determine predictors for acute Q fever in children in the general population. We retrospectively studied all children tested for *Coxiella burnetii* by serology and/or PCR upon request of their general practitioner in the regional laboratory for Medical Microbiology of the Jeroen Bosch during the Q fever outbreak in the Netherlands between 2007 and 2011. A total of 1061 patients was analyzed. Influenza-like illness and respiratory tract infection were the most common presentations of acute Q fever, mimicking other common childhood illnesses. None of the reported symptoms was significantly related to a positive test outcome and therefore presenting signs or symptoms have no predictive value in diagnosing Q-fever in children. Only diagnostic tests are reliable. As the infection generally follows a mild and uncomplicated course, we question if the difficulty of recognizing pediatric Q fever is a problem worth solving.

## Introduction

**Figure 1:**
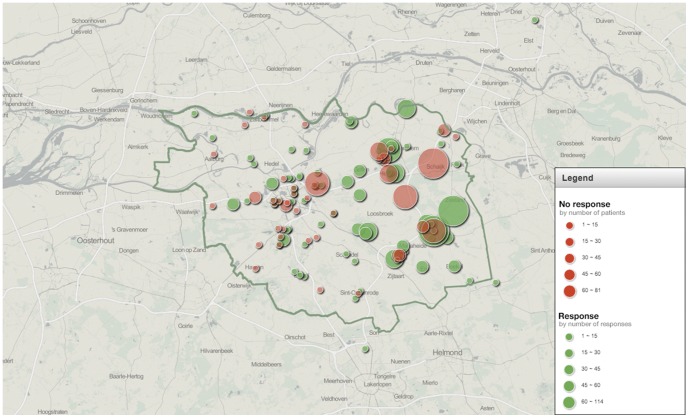
Geographical distribution and number of responders and non-responders to the questionnaire. Dark green line: Jeroen Bosch Hospital laboratory encachment area. Responders and non-responders: see legend within the image.

Between 2007 and 2011, **t**he Netherlands was confronted with the largest human Q fever outbreak ever reported; in 2009, 2,354 Q fever cases were reported to the public health authorities [1]. The outbreak was concentrated in the northeastern part of the province of Noord-Brabant (*figure 1*). During the epidemic, the Regional Laboratory for Medical Microbiology and Infection Prevention of the Jeroen Bosch Hospital was the major provider of *C. burnetii* diagnostics for this region.

Q fever usually presents as a self-limiting febrile disease [2]. It may also manifest with a variety of non-specific symptoms that are commonly encountered in other illnesses [2], or remain asymptomatic [1], [3]. Knowledge of Q fever has increased over the last decades, but research has mainly focused on adults. Data in children are scarce; current knowledge is mostly based on case reports [4]–[17] and small case series [3], [18]–[22]. Fever and pneumonia are most frequently reported in these cases, followed by central nervous system infection and myocarditis. A bias towards more severe cases seems to be likely in the available literature. As a result, selecting the right children for Q fever diagnostics in everyday practice is difficult.

We aimed to determine predictors for a positive Q fever test in children in the general population. With these predictors, the risk profile can be analyzed and used to decide whether or not tests for *C. burnetii* infection are warranted in a particular child.

In the Netherlands, the primary care system is the key entry for health care. Therefore, we studied a case series of children who visited their general practitioner during the Q fever outbreak with complaints that prompted their physician to test for *C. burnetii* infection.

## Methods

### Study population

We analyzed all data from children (0–18 years) that were tested upon request of their general practitioner for *C. burnetii* by serology and/or PCR in the regional laboratory for Medical Microbiology and Infection Prevention of the Jeroen Bosch Hospital during the Q fever outbreak from September 2007 until June 2010. The study area and period coincided with a high incidence of Q fever [23].

### Data collection

In July 2011, all general practitioners who had ordered Q fever tests in pediatric patients were asked to answer a written multiple-choice questionnaire about the child's reported symptoms at the time of sampling. Data were derived from their electronic medical records (EMR), after informed consent from the parents and – if applicable – children was obtained. The items *unexplained fever*, *lower respiratory tract infection*, *skin rash*, *vomiting - diarrhea and/or abdominal pain* and *other* could be chosen. In addition, age, gender and the outcome(s) of the test(s) were noted.

### Laboratory diagnosis of acute Q-fever

Laboratory diagnosis of acute Q-fever depends on the duration of symptoms. The first 2 to 3 weeks after the onset of disease, no antibodies are formed and diagnosis can only be made based on a positive polymerase chain reaction (PCR) [24]. After this, IgM phase I/II antibodies are the first to be detected followed shortly by IgG phase II. IgG phase I antibodies are the last to be formed, sometimes even weeks to months after the onset of infection. Therefore, anti-phase II antibodies predominate during acute Q-fever, while anti-phase I antibodies are more likely to be elevated in chronic Q-fever cases [25], [26].

All sera in this study were analyzed in the Regional Laboratory of Medical Microbiology and Infection Prevention at the Jeroen Bosch Hospital, ‘s-Hertogenbosch, The Netherlands. During the outbreak, a diagnostic screening strategy was implemented to cope with the large number of samples. This strategy combined an enzyme-linked immunoassay (ELISA) IgM phase II (Serion Immundiagnostica, Wurzburg Germany) or an in house PCR test, depending on the time after onset of symptoms, with confirmation and follow-up by an indirect immunofluorescence assay (IFA) (Focus Diagnostics, Cypress, CA, USA) [24], [25]. IFA is considered the reference method [26].

### Statistical analysis

All possible predictors were investigated by using the Fisher exact test or a t test. We used a binomial test to investigate the differences between the general practitioners in their success of predicting seropositive outcomes (using SPSS). A regression analysis with two successful predictors using mixed models with a binomial outcome, the general practitioners constituting a random factor (using the package lme4 in R) was also performed.

## Results

### Study population

All 2088 patients for whom tests were ordered by general practitioners were included in the study. A total of 1061 questionnaires were returned (50.8%). In 40 patients, the general practitioners could not retrieve the presenting symptoms from their EMR; these cases were excluded from further analysis.

### Patient characteristics

There were no differences in the distribution of age, gender and test outcome between the responder and the non-responder group (*table 1*). The geographical distribution of the general practitioners who did or did not respond to our questionnaire was similar, and equally distributed in the area of the outbreak (*figure 1*).

**Table 1:** Patient characteristics.

	Responder group	Non-responder group
Patients (number)	1021	1027
Mean age (years)	11,69	11,52
Male gender (percent)	47,4	46,0
Acute Q-fever present (percent)	4,8	4,3
General practitioners (number)	141	99

**Figure 2:**
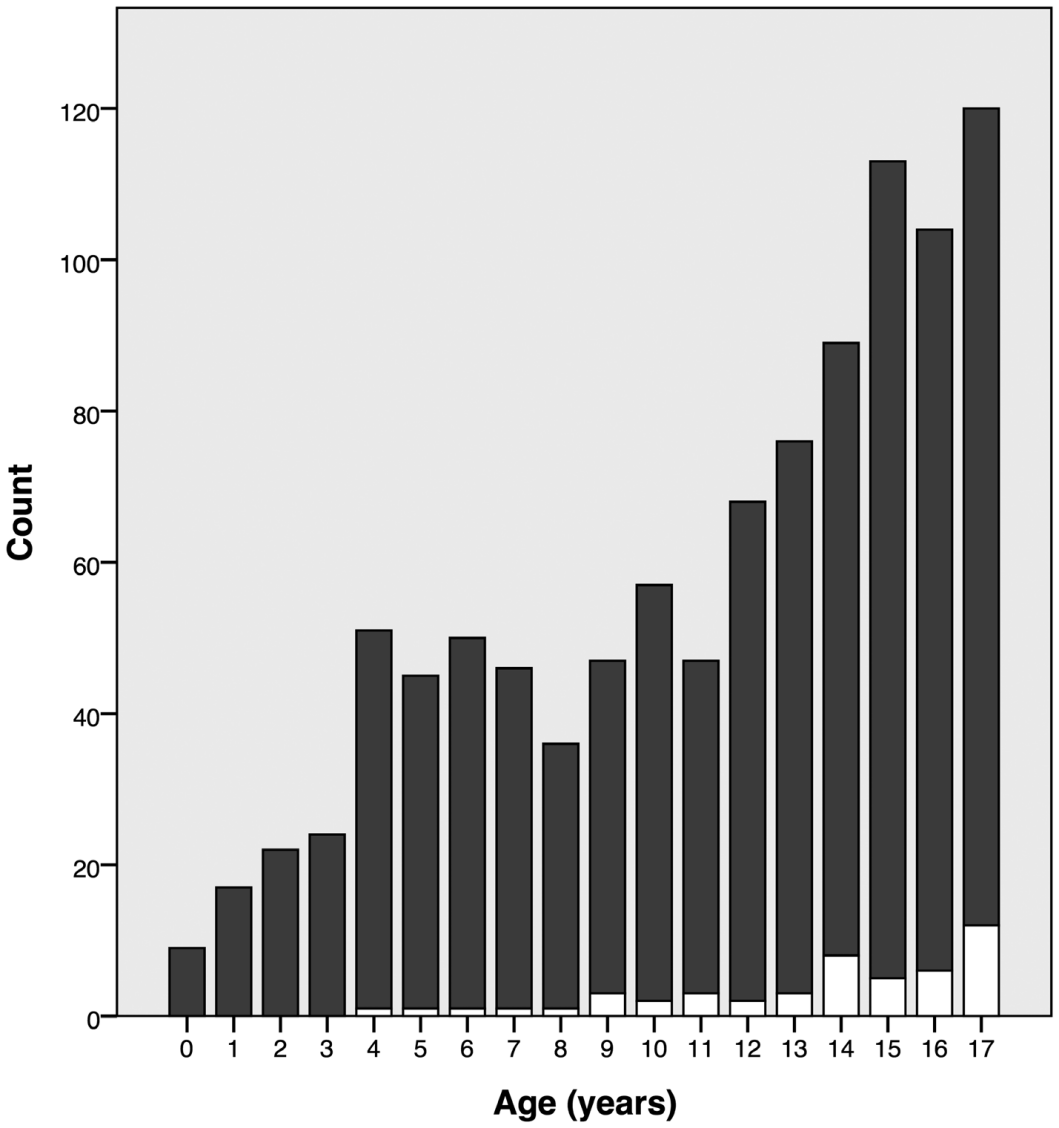
Number of requested tests and test outcome divided per age. White bar: acute Q fever. Dark grey bar: no acute Q fever.

Of the 1021 children analyzed, 484 were boys, of which 28 were diagnosed with acute Q fever, and 537 were girls, of which 21 were diagnosed with acute Q fever. No significant relation between gender and a positive test result was found (chi square [1, N = 1021] = 1.958, p = 0.162). The age of the children ranged between 0.28 and 17.98 years (M 11.7; SD 4.7); the number of children tested increased with increasing age (*figure 2*).

Children with a positive test were older (positive test M 13.9 years, SD 3.6; negative test M 11.6 years, SD 4.7; no homogeneity of variance, Levene's test, F = 13.104, p = 0.000; Welch t test (57) = 4.337, p = 0.000). This is not merely caused by more tests being performed in older children; the proportion of diagnosed infections is also significantly related to age (r = 0.108, N = 1021, p = 0.001).

### Reported symptoms

The original answers to the multiple-choice question are shown in table 2. Most patients presented with more than one symptom (mean 1.8 symptoms per patient). The top 5 reported symptoms in confirmed acute Q-fever cases were fatigue (n = 22), lower respiratory tract infection (n = 13), cough (n = 13), headache (n = 9), and fever (n = 8). O*ther* was the multiple-choice item that was most often reported. When the item *unexplained fever* had been chosen, often other symptoms had been added to *other* in writing that could explain the fever, however. In those cases, we reclassified *unexplained fever* to *respiratory tract infection*, *gastro-intestinal tract infection* or *influenza-like infection* as appropriate. The remarks written under the item *other* were grouped as follows (*table 3*): *upper respiratory tract infection* (i.e. cough, aching throat, pharyngitis, tonsillitis, sinusitis, rhinitis, ear pain, cervical lymphadenopathy); *lower respiratory tract infection* (i.e. symptoms described as under *upper respiratory tract infection* but combined with either tachypnea or dyspnea); *undetermined respiratory tract infection* (i.e. single presenting symptoms such as dyspnea or tachypnea); *influenza-like infection* (i.e. 2 or more of: fever, headache, nausea, malaise, myalgia, arthralgia or upper respiratory tract infection with one or more of the above mentioned symptoms excluding fever); *malaise* (i.e. bad thrive, malaise, faint, or tiredness); *Q fever in the direct vicinity of the patient* (i.e. visited a farm with known Q fever, family member or close contact with Q fever).

**Table 2:** Reported items in the original multiple-choice questionnaire.

Items	Reported (number)
Unexplained fever	193
Lower respiratory tract infection	263
Skin rash	5
Vomiting, diarrhea, and/or abdominal pain	122
Other	1310
Total	1893

**Table 3:** Modified groups of reported symptoms.

Modified groups	Reported cases in the group without acute Q fever (%) (n = 972)	Reported cases in the acute Q fever group (%) (n = 49)
Lower respiratory tract infection	249 (25,6)	12 (24,5)
Upper respiratory tract infection	183 (18,8)	10 (20,4)
Undetermined respiratory tract infection	25 (2,6)	2 (4,1)
Unexplained fever	94 (9,7)	2 (4,1)
Skin rash	5 (0,5)	0
Vomiting, diarrhea, abdominal pain or gastroenteritis-like symptoms	93 (9,6)	4 (8,2)
Influenza-like symptoms	220 (22,6)	12 (24,5)
Malaise	126 (13,0)	8 (16,3)
Q-fever in the direct vicinity of the patient	51 (5,2)	4^1^(8,2)
Other^2^	59 (6,1)	2 (4,1)
Total	1105	56

1 As single ‘symptom’ in 2 patients.

2 *Other* contains: patient's request, profuse sweating, myalgia/arthralgia, headache, dizziness, palpitations, sleeping difficulty and weight loss; total number of symptoms in the non-acute Q fever group is 62, three cases having two symptoms.

For explanation of the modification see text.

49 children (4.8%) in the response group were diagnosed with acute Q fever at the time of laboratory testing, but none of the reported presenting symptoms was significantly related to a positive test outcome. No symptoms were significantly related to a negative test outcome either. None of the Fisher exact tests applied to the group of modified symptoms in Table 3 returned a significant outcome (the lowest p value, 0.310, was far above the alpha level of 0.05). None of the modified reported symptoms had a relationship with the presence of acute Q fever; in other words, no predictors for positivity could be identified among the reported presenting symptoms.

Two patients had no disease manifestations but were tested because of Q fever in their direct vicinity. This number is too small for statistical analysis of its predictive value.

### General practitioner characteristics

The distance between the epicenter of the epidemic and the practice of the general practitioner ranged between 0.25 and 38.45 kilometers (M = 18.73, SD = 8.27). The number of tests per general practitioner was correlated to the distance of the practice to the epicenter, but the correlation coefficient was moderate (r = −0.283, N = 140, p = 0.001). There were also several general practitioners close to the epicenter who did not request many tests, and some having their practice much further away who did.

There is a significant correlation between distance and positive diagnosis (r = −0.072, N = 1021, p = 0. 021). The overall probability of a positive outcome was fairly low for all general practitioners: 4.8% (49 positive outcomes on 1021 tests), but that does not exclude differences in performance between them. Five of the 140 practitioners performed beyond the average level (one-directional binomial test, criterion being a p value of 0.048), a number that is within the range of more extreme performances to be expected purely on the basis of probability. An important observation is that all five practitioners had their practice located near the epicenter.

### A regression model to predict acute Q fever

In order to identify factors that needed to be included to predict the probability of a positive test, we applied regression analysis, with general practitioners as a random effect (generalized linear mixed model analysis; lme4, package R). The null model in the analysis contains no effects, only including general practitioners as a random effect. The Akaikes Information Criterion (AIC) of this model was 388.86. Further analyses clarified that both the age of the children and distance from the epicenter play a significant role in predicting a positive test outcome. Testing several models of how to apply these variables, the best implementation (lowest AIC) was a squared value for age (in years) and a categorical distance parameter, distinguishing between a distance of 15 kilometers or less from the epicenter and a distance of more than 15 kilometers. The resulting model has an AIC of 375.19, a clear improvement compared to the null model. Both variables were significant (age squared, z = 3.087, p = 0.002; distance, two groups, z = −2.492, p = 0.013). Including these two variables had the effect that no individual practitioners performed significantly better any longer in their test outcome prediction (their normalized random intercept values were all between −1 and 1).

## Discussion

Our study shows that it is difficult to accurately identify acute Q fever in children based on signs and symptoms alone. They appear to have no predictive power. Clinicians have to rely on diagnostic tests to confirm or reject the diagnosis. On one hand, this can lead to large numbers of children being tested during an epidemic, with large numbers of negative test results, posing a great burden on the available laboratory facilities. On the other hand, Q fever may be easily missed outside an epidemic. Consistent with earlier reports, respiratory tract infection and influenza-like illness were the most frequently reported presentation in acute Q fever [1], [3], [18]. These clinical presentations are most often caused by other pathogens in children. Given the frequent mild and self-limiting course of illness, investigations to define the causative organism are generally not undertaken. It is doubtful whether this is an important clinical problem, since our data also indicate that Q fever is generally mild and uncomplicated in children: there were no children who were diagnosed with chronic Q-fever during our study, and no serious acute illness requiring hospital admission was reported.

Two variables turned out to be predictive of a positive test result: the distance to the epicenter of the epidemic, and the age of the tested child. The best prediction was a squared value for age (years) and a two-valued distance parameter (living <15 or >15 kilometers from the epicenter). Once these predictors were taken into account in the data analysis, no differences were found between the general practitioners in their test outcome performance. None of them was able to increase their efficacy in making a correct clinical diagnosis based on signs and symptoms alone.

An increased incidence of acute Q fever with increasing age has been reported before, and has been explained by increased exposure to *C. burnetii* when children get older, similar to the increased risk of acute Q fever with occupational exposure in adults [18], [27]. The recent Dutch epidemic originated from goat farms, and residency or performing recreational activities in the neighborhood of these farms were determined to be important risk factors for infection, probably due to a combination of wind and dry weather [28]. It is not very likely that this kind of exposure increases with increasing age. It has also been suggested that young children are presented less to a general practitioner, as they often remain asymptomatic during an acute Q fever infection [29]. However, even after correcting for this effect, the percentage of positive test outcomes was still correlated to increasing age in our study, which renders this explanation unlikely.

A relationship between the risk of infection and the distance from the infectious source has been reported before. In a recent Dutch study on 96 adults in 2008, residency within 2 km from the source farm greatly increased the risk for infection, whereas residency at a distance of 5 km or more from the source farm seemed protective [30]. The epicenter in our study comprised of multiple affected dairy goat farms distributed over a larger area, and we estimated the distances from the epicenter based on the location of the general practitioner, which makes distance a less precise parameter in our study. Nevertheless, we found a sharp boundary in relation to infection as well, indicating that distance is a direct and determining factor in an acute Q fever epidemic.

Our study poses several limitations. Firstly, we used passive surveillance to determine the predictors of acute pediatric Q fever, creating the possibility of selection bias.

Performing a prospective cohort study would have eliminated this factor because the decision to test for Q fever would no longer depend on the individual general practitioner. Unfortunately, this is so labor intensive and expensive to perform that it is not feasible. The national Q fever epidemic gave us the opportunity to obtain as reliable data as possible. Secondly, our study only included the patients tested by their general practitioner, it is possible that severely sick children are not represented in our data because they were directly referred to the hospital, and primarily tested there. Maltezou et al. reported a low hospitalization rate in pediatric acute Q fever, however, which suggests that such ‘direct referrals’ are rare [3]. Also, only one pediatric patient was primarily diagnosed with acute Q fever in the Jeroen Bosch Hospital during the study period (encachment area in relation to the epidemic: see figure 1). The patient presented with an influenza-like illness and the course of illness was uncomplicated.

To our knowledge, our study presents the largest specific case series of acute Q-fever in children. We conclude that pediatric acute Q fever mimics other common respiratory and/or influenza-like illnesses, and is clinically difficult to distinguish from other pathogens. Fortunately, pediatric Q fever generally follows a mild and uncomplicated course. We therefore question if the difficulty of recognizing pediatric Q fever is an important problem that is worth solving.
